# TGF-β1, Released by Myofibroblasts, Differentially Regulates Transcription and Function of Sodium and Potassium Channels in Adult Rat Ventricular Myocytes

**DOI:** 10.1371/journal.pone.0055391

**Published:** 2013-02-05

**Authors:** Kuljeet Kaur, Manuel Zarzoso, Daniela Ponce-Balbuena, Guadalupe Guerrero-Serna, Luqia Hou, Hassan Musa, José Jalife

**Affiliations:** Center for Arrhythmia Research, University of Michigan, Ann Arbor, Michigan, United States of America; Università degli Studi di Milano, Italy

## Abstract

Cardiac injury promotes fibroblasts activation and differentiation into myofibroblasts, which are hypersecretory of multiple cytokines. It is unknown whether any of such cytokines are involved in the electrophysiological remodeling of adult cardiomyocytes. We cultured adult cardiomyocytes for 3 days in cardiac fibroblast conditioned medium (FCM) from adult rats. In whole-cell voltage-clamp experiments, FCM-treated myocytes had 41% more peak inward sodium current (I_Na_) density at −40 mV than myocytes in control medium (p<0.01). In contrast, peak transient outward current (I_to_) was decreased by ∼55% at 60 mV (p<0.001). Protein analysis of FCM demonstrated that the concentration of TGF-β1 was >3 fold greater in FCM than control, which suggested that FCM effects could be mediated by TGF-β1. This was confirmed by pre-treatment with TGF-β1 neutralizing antibody, which abolished the FCM-induced changes in both I_Na_ and I_to_. In current-clamp experiments TGF-β1 (10 ng/ml) prolonged the action potential duration at 30, 50, and 90 repolarization (p<0.05); at 50 ng/ml it gave rise to early afterdepolarizations. In voltage-clamp experiments, TGF-β1 increased I_Na_ density in a dose-dependent manner without affecting voltage dependence of activation or inactivation. I_Na_ density was −36.25±2.8 pA/pF in control, −59.17±6.2 pA/pF at 0.1 ng/ml (p<0.01), and −58.22±6.6 pA/pF at 1 ng/ml (p<0.01). In sharp contrast, I_to_ density decreased from 22.2±1.2 pA/pF to 12.7±0.98 pA/pF (p<0.001) at 10 ng/ml. At 1 ng/ml TGF-β1 significantly increased *SCN5A* (Na_V_1.5) (+73%; p<0.01), while reducing *KCNIP2* (*Kchip2;* −77%; p<0.01) and *KCND2* (K_V_4.2; −50% p<0.05) mRNA levels. Further, the TGF-β1-induced increase in I_Na_ was mediated through activation of the PI3K-AKT pathway via phosphorylation of FOXO1 (a negative regulator of *SCN5A*). TGF-β1 released by myofibroblasts differentially regulates transcription and function of the main cardiac sodium channel and of the channel responsible for the transient outward current. The results provide new mechanistic insight into the electrical remodeling associated with myocardial injury.

## Introduction

Cardiac fibroblasts represent the most abundant cell type in the heart. [1] Myocardial disease promotes fibroblast proliferation and differentiation into myofibroblasts, which are hypersecretory of both ECM components and multiple cytokines and growth factors that are known to affect myocyte morphology and function. [2] Cardiac fibroblast conditioned medium (FCM) from neonatal rats has been shown to protect cardiac myocytes against hypoxia induced injury in isolated cells and the growth factors in such medium also had a protective effect against ischemia-reperfusion injury. [3] Protein analysis of the neonatal rat FCM has shown that these cells release proinflammatory cytokines, such as transforming growth factor β-1 (TGF-β1), fibroblast growth factor (FGF), interferon-γ (IFN-γ), cytokine-induced neutrophil chemoattractant (CINC-1), macrophage migration inhibitory factor (MIF), vascular endothelial growth factor (VEGF) and tumor necrosis factor, [2] many of which are significantly upregulated after myocardial injury. [4], [5] Neonatal rat fibroblast also release significant amounts of IGF-1, endothelin A and leukemia inhibitory factor proteins, which are some of the proteins responsible for hypertrophy in cardiac myocytes and increased collagen synthesis in fibroblasts due to FCM. [6], [7].

A number of cytokines may affect the electrical properties of cells. Overnight incubation of rat sensory neurons with chemokine (C-X-C motif) ligand 1 (CXCL1) increased both TTX-resistant and TTX-sensitive sodium currents (I_Na_). [8] CXCL1 also increased voltage-activated K current densities [9]. In rat models of myocardial infarction, mRNA levels of TGF-β1 [10] are increased up to 50-fold in the infarcted area and up to 15 fold in the non-infarcted myocardium [1], [5], [11]. Early reports on the consequences of culturing neonatal myocytes in cardiac FCM showed increased protein expression, [12] action potential duration (APD) prolongation and K_V_4.2 downregulation. [13] Pedrotty et al, [14] have shown that exposure of neonatal rat ventricular myocyte (NRVM) monolayers to FCM produced dose-dependent reduction in conduction velocity, prolongation of APD, depolarization of the resting membrane potential and reduction of the AP upstroke velocity. Fibroblast proliferation, myocyte apoptosis and Cx43 expression were not affected. [14] However mRNA levels of Na_V_1.5, Kir2.1 and K_V_4.3 were all reduced by the exposure to the FCM. [14] More recently, Vazquez et al [15] showed in NRVM monolayers treated with FCM harvested from infarcted hearts that conduction velocity could be higher or lower than in NRVM treated with FCM from normal hearts, depending on cell density. In addition, the optical APD_70_ was slightly shorter in the former than in the latter. [15].

While the above effects of neonatal fibroblasts on NRVMs are of interest, they should not be extrapolated to the adult heart, particularly those derived from the myocardial infarction scar. In fact, there is evidence in the literature suggesting that the phenotypic changes produced by paracrine factors released by cardiac fibroblasts may be different in the developing versus adult myocyte. [16] In addition, while the use of FCM is of importance, the identity of the specific cytokines producing the electrophysiological changes remains unclear. Finally, despite the potential importance of both electrical and structural remodeling in ischemic heart disease, a role for specific regulation by cytokines released from cardiac fibroblasts on cardiac myocyte electrical function and/or excitation-contraction coupling has not been established in either the normal or diseased adult heart. Therefore, we have begun to investigate in adult ventricular myocytes the effects of specific cytokines that are known to be released by myofibroblasts and upregulated in the infarcted ventricle. [11], [17], [18] Here we have focused on the effects of TGF-β1 because our results provide strong evidence that this cytokine may be primarily responsible for the effects produced by FCM on the functional expression of two major ion channels of cultured adult ventricular myocytes. We have also explored the specific intracellular signaling pathways that mediate the TGF-β1-induced changes in myocyte ion channel protein functional expression and electrophysiological properties. Our working hypothesis is that TGF-β1 differentially affects cardiac sodium and potassium ion channel function by targeting signaling pathways that modify transcription and gene expression.

## Methods

### Ethics Statement

Animal care was supervised by the University of Michigan Unit for Lab Animal Medicine and all animal study protocols were approved by the University Committee on Use and Care of Animals (UCUCA).

### Adult Cardiomyocytes and Fibroblast Isolation

**Cardiomyocytes** were isolated from normal adult male CD rats (200–300 g). Briefly, after quick removal, hearts were washed in ice-cold phosphate buffered saline (PBS), then retrogradely perfused through the aorta for up to 5 minutes with modified Krebs buffer (KHB) containing (in mM) NaCl 118, KCl 4.8, HEPES 25, K_2_HPO_4_ 1.25, MgSO_4_ 1.25, glucose 11, CaCl_2_ 1, pH 7.40. The perfusate was then switched to modified Krebs buffer without calcium for 3 minutes. Following calcium-free KHB perfusion hearts were digested by perfusing calcium-free KHB containing 200 units/ml collagenase II, (Worthington Biochemicals, Lakewood, NJ) and blebbistatin (33.3 µM) for 15 min. The collagenase digested hearts were removed from the apparatus and atria were discarded. Ventricles cell suspension was centrifuged (500×*g*) for 30 sec, the cell pellet was resuspended in KHB-A containing 2% bovine serum albumin and blebbistatin. The cell suspension was centrifuged again and resuspended in culture media (Medium 119, Sigma) containing glutathione (10 mM), NaHCO_3_ (26 mM), 100 units/ml penicillin, 100 µg/ml streptomycin and 5% fetal bovine serum. Cells were plated on laminin coated (40 µg/ml) tissue culture cover slips. After 2 hr, the medium was changed to serum-free MI99.

#### Cardiac fibroblast isolation

Ventricles cell suspension supernatant from both spins was saved for fibroblast isolation. The suspended fibroblasts were centrifuged at 2000 rpm for 10 min and the cell pellet was suspended in DMEM supplemented with 1% penicillin/streptomycin, and 10% fetal bovine serum (full medium). Cardiac fibroblasts were grown in this same full medium until 70–80% confluent and passaged using 0.05% trypsin EDTA.

#### Collection of fibroblast conditioned medium

Cardiac myofibroblasts at passage 3–5 were plated in 100-cm dishes (5×10^5^). Cells were allowed to grow in full medium for one day. At the end of the growth period full medium was aspirated and cells were rinsed with Ca^2+^/Mg^2+^ free PBS and 10 ml of serum free medium was added to each dish. After 24 hr the conditioned medium was collected, filter sterilized and stored at −80°C until further used.

### Cytokine Array and TGF-β1 ELISA

Cytokine array was performed using a commercially available Proteome Profiler Rat Cytokine Array Kit (R and D systems, Minneapolis, MN). Briefly array membranes were incubated with 2 ml of conditioned medium overnight in the cold room and the assay was performed according to manufacturer’s instructions.

Levels of total TGF-β1 released in the culture medium were analyzed using commercially available Enzyme-linked immunosorbent assay kit (R and D systems, Minneapolis, MN). Briefly 2 ml of conditioned medium was activated with HCl, 100 µL of activated conditioned medium was used in the TGF-β1 ELISA kits according to manufacturer’s instructions. All assays were done in duplicate. Results are expressed as picogram of TGF-β1/ml of media.

### Cell Treatment

Isolated adult rat cardiac myocytes were treated with FCM or TGF-β1 (R and D systems, Minneapolis, MN) for 3 days in serum free medium. For PI3K pathway inhibition cells were pretreated (30 min) with 10 µM LY29004 (Cayman Chemicals, Ann Arbor) before the treatment with TGF-β1.

### mRNA Analysis by Quantitative PCR (qPCR)

Cardiac myocytes were washed with PBS and lysed with lysis buffer. RNA was isolated from the myocardial tissue using RNAeasy kit from Qiagen (Qiagen, Valencia, CA) according to the manufacturer’s instructions. Isolated RNA from these samples was treated with DNase for 15 min at room temperature (Qiagen, Valencia, CA). 100 ng of DNA-free total poly-A tail RNA (mRNA) was first subjected to synthesis of cDNA using Oligo dT primers applying SuperScript III First-Strand Synthesis System from Invitrogen (Invitrogen, Carlsbad, CA) according to the manufacturer’s instructions. cDNA from 20 ng of total RNA was then subjected to real-time RT-PCR using predesigned taqman probe primers specific for rat *Scn5a* (Rn00565502), *Kcnip2* (Kchip2; Rn01411451) and kcnd2 (Rn01456260) (Applied Biosystems, California). No-template controls and no-RT controls were run during each experiment to detect any RNA and/or DNA contamination. Results are expressed as fold expression of gene of interest normalized to GAPDH expression in the sample.

### Western Blotting

Control and treated cardiac myocytes were washed in cold PBS, lysed directly in the modified loading buffer (25 mmol/l Tris•HCl; 150 mmol/l NaCl; 1 mmol/l EDTA; 4 mmol/l NaF; 2 mmol/l Sodium ortho-vanadate; 1% Triton X-100, protease inhibitor, 5% glycerol, 1%SDS, 0.05%bromophenol blue, 5% β mercaptoethanol) and sonicated. The lysate (20 µl) were then subjected SDS-PAGE as described earlier. The blots were incubated with rabbit pFOXO1 antibody, 1∶500 (Cell Signaling, ) or rabbit GAPDH antibody, 1∶5000 (Sigma-Aldrich, St. Louis, MO).

### Patch-clamp Experiments

Whole-cell ionic currents were recorded from adult rat ventricular myocytes. All recordings were conducted at room temperature.

**Sodium current** recordings were conducted in a low-sodium extracellular solution containing (in mM): NaCl, 10; MgCl_2_, 1; CaCl_2_, 1.8; CdCl_2_, 0.1; HEPES, 20; CsCl, 127.5; glucose, 11. The pipette solution contained (in mM): NaCl, 5; CsF, 135; EGTA, 10; MgATP, 5; HEPES, 5. To characterize the voltage dependence of the peak I_Na_, single cells were held at −120 mV, and 200 msec voltage steps were applied from −90 to +30 mV in 5 mV increments. The interval between voltage steps was 3 sec. Voltage-dependence of inactivation was assessed by holding cells at various potentials from −160 to −40 mV followed by a 30 msec test pulse to −40 mV to elicit I_Na_. Recovery from inactivation was studied by holding cells at −120 mV and applying two 20-msec test pulses (S1, S2) to −40 mV separated by increments of 2 msec to a maximum S1–S2 interval of 80 msec. The S1–S1 interval was kept constant at 3 sec.

The extracellular solution for **transient outward potassium current (I_to_)** contained (in mM): 136 NaCl, 4 KCl, 1.8 CaCl_2_, 2 MgCl_2_, 10 HEPES, 0.03 tetrodotoxin, 0.01 nifedipine, and 14 glucose, pH 7.35. The pipette solution contained (in mM):135 KCl, 1 MgCl_2_, 10 EGTA, 10 HEPES, 5 glucose, pH 7.2. Voltage-gated outward K^+^ currents were evoked during 5-s depolarizing voltage steps to potentials between −40 and +60 mV from a holding potential of −70 mV; voltage steps were presented in 10 mV increments at 15 s intervals.

**Action potentials** were recorded from individual myocytes using the current clamp mode of the MultiClamp 700 B amplifier after gigaseal formation and patch break. Stimulus pulses (1–2 ms duration) were generated using a World Precision Instruments DS8000 stimulator (Sarasota, FL). The bath solution contained (in mM): NaCl: 148, KCl: 5.4, MgCl_2_: 1, CaCl_2_ 1.8, NaH_2_PO_4_: 0.4, HEPES: 15, Glucose: 5.5, pH 7.4 with NaOH. The pipette solution contained (in mM), KCl: 20, K-aspartate: 90, KH_2_PO4: 10, EDTA: 5.0, K_2_ATP: 1.9, HEPES: 5.0 and Mg^2+^7.9; pH 7.2 (KOH).

### Statistical Analyses

**Figure 1:**
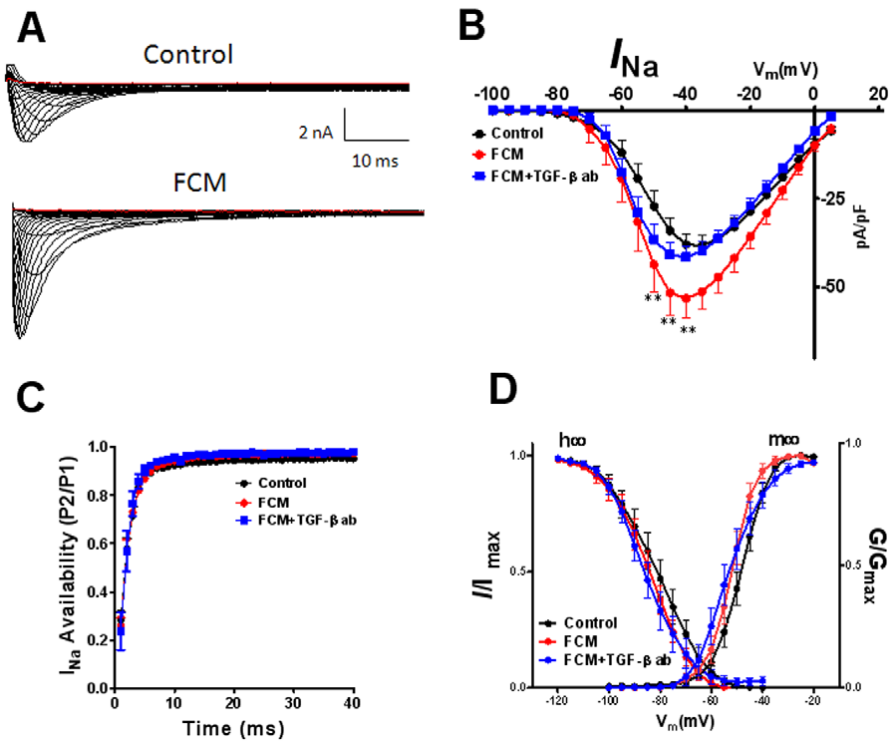
Effects of fibroblast conditioned medium (FCM) on peak inward sodium current (I_Na_) after 72 hr of treatment. Representative current traces A) Top Panel, control; Lower Panel, FCM. Current-voltage relationships for control (black), FCM (red) and FCM+TGF-β1 antibody (TGF-β1 ab) (blue) is shown in panel B. C) Time dependent recovery of the channel. D) Voltage dependence of inactivation (h∞ curve) and Voltage dependence of activation (m∞ curve). Values are mean ± standard error of the mean N = 13−29 cells from 7 different isolations. **indicates p<0.01 significant difference between control and FCM treated cells by two way ANOVA with Bonferroni post-test.

In all cases, “N” indicates the number of animals and “n” the number of experiments (e.g., Fig. 1). Comparisons of individual group means used a two-tailed Student’s t test. One-way analysis of variance (ANOVA) with Bonferroni post-test was used to compare multiple data sets. All statistical calculations were done using GraphPad Prism version 5 (GraphPad Software Inc., San Diego, Calif.) and p<0.05 was considered significant. Data are presented as mean ± standard error of the mean.

## Results

In all experiments, cells were exposed to one of the following for 72 hrs prior to the experiment: control medium, FCM or TGF-β1.

In control experiments the mean peak sodium current measured in freshly dissociated rat ventricular myocytes (Day 0) was similar to that measured after 72 hours in control medium (Day 3). At −45 mV, peak I_Na_ was −38.15±1.79 pA/pF in Day 0 cells (n = 3) and −36.75±4.53 pA/pF in Day 3 cells. (n = 5). This difference was not statistically significant, which agrees with previous work from our lab showing no difference in sodium channel properties between days 0 and 5 in culture. [19].

### Exposure of ARVMs to FCM Increases Sodium Current

Fig. 1 shows data from whole cell-voltage clamp experiments in which I_Na_ was recorded at room temperature, in a low-sodium extracellular solution (see [20] for details) and holding potential (HP) of −120 mV; 200 msec voltage steps were applied from −90 to +30 mV in 5 mV increments every 3 sec.Fig. 1 A shows representative superimposed I_Na_ traces obtained at varying voltages in control top panel and FCM lower panel Surprisingly, I_Na_ for FCM was larger than control at all voltages. B shows superimposed mean I_Na_ current density-voltage (IV) relations. Compared to control (black), incubation for 3 days with FCM (red) significantly increased peak current at voltages between −50 and −40 mV (p<0.01). Voltage-dependence of activation (m∞) and inactivation (h∞) was assessed by holding cells at various potentials from −160 to −40 mV followed by a 30 msec test pulse to −40 mV to elicit I_Na_. [20] As illustrated in Panels D, neither the h∞ nor the m∞ curve was modified, suggesting that FCM did not change the channel’s biophysical properties.

### Exposure of ARVMs to FCM Reduces the Transient Outward Current

**Figure 2:**
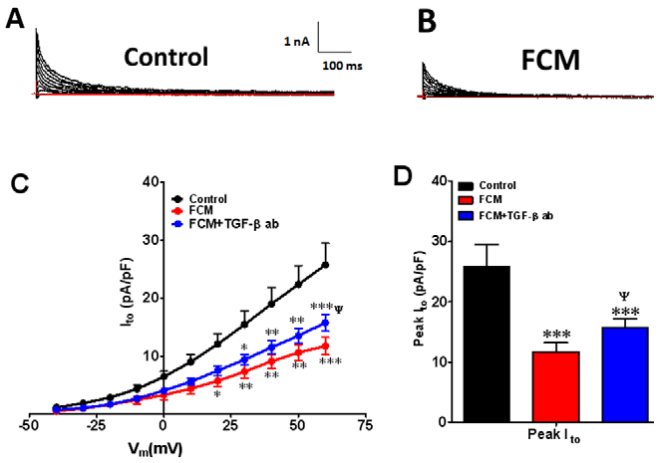
Effects of fibroblast conditioned medium (FCM) on outward potassium current (I_to_) after 72 hr treatment. Representative current traces A) control, B) FCM. I_to_ IV relation at voltages between −40 and +60 mV for control (black), FCM (red) and FCM+TGF-β1 ab (blue) is shown in panel C. D) peak I_to_ under control conditions. Values are mean ± standard error of the mean. N = 10 cells from 5 different isolations. *indicates p<0.05, **indicates p<0.01, ***indicates p<0.001 significant difference between control and FCM treated cells. Δ indicates p<0.05 significant difference between FCM+TGF-β1 ab and FCM treated cells.

Next, we investigated the effects of FCM on I_to_ to determine whether the downregulation of K_V_4.2 reported by other authors in neonatal rat myocytes, [13] as well as some heart failure models, [21] applies also to adult myocytes. Fig. 2 summarizes data conducted at room temperature. The top panels show representative superimposed I_to_ traces in cultured ARVMs obtained at varying voltages in the absence (A) and the presence of FCM (B). Three day exposure to FCM reduced I_to_ with respect to control at all voltages. Panel C shows, the superimposed I_to_ IV relations. Incubation of rat adult cardiac myocytes for 3 days with FCM (red) significantly decreased peak I_to_ at voltages between 20 and 60 mV compared to the control treated cells (black) (p<0.05−0.001). For example, at 60 mV current in control cells was 25.8±3.7 pA/pF whereas in FCM it was 11.7±1.4 pA/pF (p<0.001).

#### Cardiac myofibroblasts release active proteins in the culture medium

**Figure 3:**
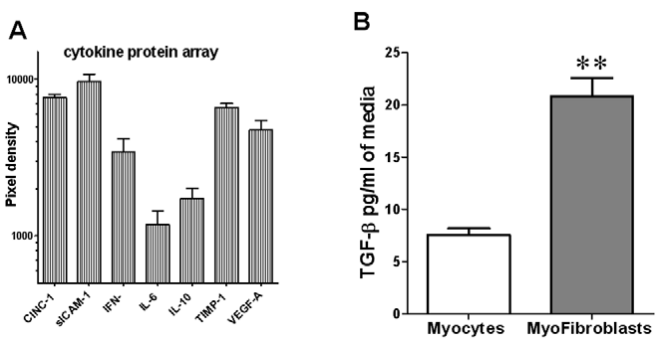
Protein analyses of cytokine expression in fibroblast conditioned media. 3A) Protein analyses of cytokine expression in fibroblast conditioned media by cytokine array. Values are mean ± standard error of the mean, N = 3 in each group. 3B). Concentration of TGF-β1 in rat myocyte conditioned medium (black) and FCM (white) determined using a rat TGF-β1 ELISA kit (R&D Systems). Values are mean ± standard error of the mean, N = 3 in each group, **indicates p<0.01 significant difference between myocyte and FCM levels.

The unexpected results presented above suggest that cardiac fibroblasts introduced a soluble factor or factors into the medium that differentially altered I_Na_ and I_to_ current densities by either *1*) a direct cell membrane or intracellular interaction; or *2*) sequestration, consumption, or modification of factors in the standard medium, leading to an indirect biological effect. [2] Thus, in an initial attempt to address those questions, we have used 1 ml FCM collected from fibroblasts harvested from normal rat hearts for analysis of specific cytokine proteins using a rat cytokine array kit. In Fig. 3A relative quantification of relevant cytokine proteins showed that CINC-1, sICAM-1, IFN-γ, IL-6, IL-10, TIMP-1 and VEGF were present at high levels in FCM as compared to myocytes conditioned media (Fig. 3A). Note that TGF-β1, whose active form measurement requires an acidic medium, is excluded from this array because acidification is likely to affect quantification of the other cytokines. Thus, we used a rat TGF-β1 ELISA kit to determine TGF-β1 levels in FBS-free supernatant of cultured fibroblasts harvested from the ventricles of a normal rat. As shown in Fig. 3B the concentration of TGF-β1 in FCM was almost 3 times larger than that measured in myocytes conditioned medium (7.5±0.1 vs 20.8±1.7 pg/ml, p<0.01). All other cytokines shown in Fig, 3A were also detected in myocyte conditioned medium. But with the exception of CINC-1, their levels were significantly lower than in FCM.

### FCM Effects are Inhibited by a Neutralizing TGF-β1 Antibody

We conducted additional voltage clamp experiments to determine whether the differential FCM-induced changes in I_Na_ and I_to_ in our study depend, at least in part, on the TGF-β1 that is present at large concentration in that medium. Interestingly, as shown in Fig. 1B, 3-day incubation with FCM plus a TGF-β1 neutralizing antibody (FCM+, TGF-β1 ab blue), completely eliminated the FCM induced increase in peak I_Na_ current. Panel C shows that the recovery from inactivation was unaffected by the FCM or the antibody. On the other hand, as shown in Fig. 2, 3-day incubation with FCM plus a TGF-β1 neutralizing antibody (FCM+ TGF-β1 ab blue) partially prevented the FCM effect on I_to_. At 60 mV, current in FCM+ TGF-β1 ab was 15.8±1.5 pA/pF, a value that was intermediate between control and FCM (p<0.05 when comparing FCM+ TGF-β1 ab vs FCM alone).

### TGF-β1 Increases Sodium Current in Adult Cardiac myocytes

**Figure 4:**
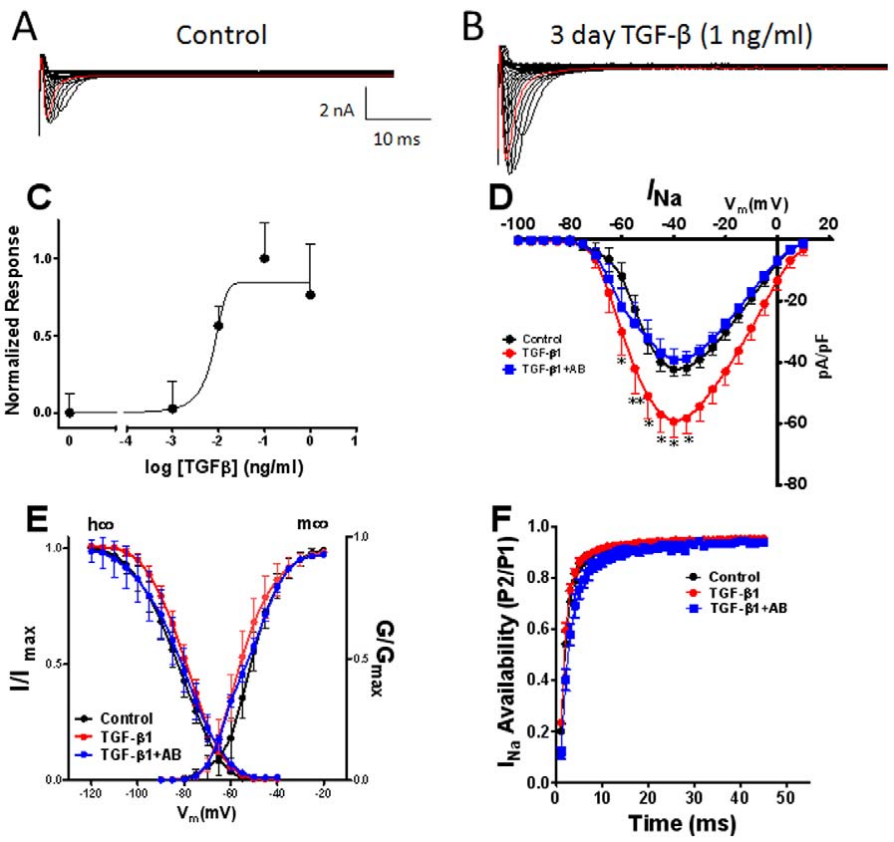
Effects of TGF-β1 on peak inward sodium current (I_Na_) after 72 hr of treatment. Representative current traces A) control, B) TGF-β1. C). Dose response curve. Data are from 61 cells, 24 hearts. D) Current-voltage relationships for control (black) TGF-β1 (red) and TGF-β1+TGF-β1 antibody (AB) (blue). Shown in panel E, Voltage dependence of activation (m∞ curve) and inactivation (h∞ curve). F) Time dependent recovery of the channel. Values are mean ± standard error. N = 12 cells from 6 different isolations. *indicates p<0.05, **indicates p<0.01 significant difference between control and treated cells.

The results presented in the previous sections suggest that TGF-β1 is a major cytokine involved in the differential FCM-induced changes in I_Na_ and I_to_. We tested this hypothesis by conducting additional patch-clamp experiments to determine whether incubation of ARVM with exogenous TGF-β1 alone would modify I_Na_ and I_to_ densities. The superimposed current traces presented in Fig. 4A and B were obtained from a representative experiment. Clearly, 3-day exposure to 1 ng/ml TGF-β1 increased the current magnitude at all voltages. Fig. 4C shows a dose-response curve for peak inward I_Na_ density obtained when culturing myocytes for 72 hours in medium containing varying concentrations (0.001−1.0 ng/ml) of TGF-β1. A maximum I_Na_ increase of ∼40% was achieved at 1 ng/ml, which was also seen at 10 ng/ml (data not shown). The calculated EC50 was 0.007 ng/ml; i.e., well below the TGF-β1 concentration in FCM (∼21 pg/ml; see Fig. 3B). As shown by the IV relation in panel D, 1 ng/ml TGF-β1 significantly increased the peak I_Na_ density at step voltages between −60 mV and −30 mV (p<0.05−0.01). At −40 V the TGF-β1 treated cells had about 40% more inward current compared to cells treated with control medium (p<0.05). Most important, the TGF-β1 induced changes in I_Na_ peak density were completely abolished when cells were treated with TGF-β1 plus the neutralizing TGF-β1 antibody, demonstrating the specificity of the TGF-β1 signaling effects. Finally, as illustrated in Fig. 4E, neither the m∞ nor the h∞ curve was modified by TGF-β1 treatment.

### TGF-β1 Decreases I_to_ in Adult Cardiac Myocytes

**Figure 5:**
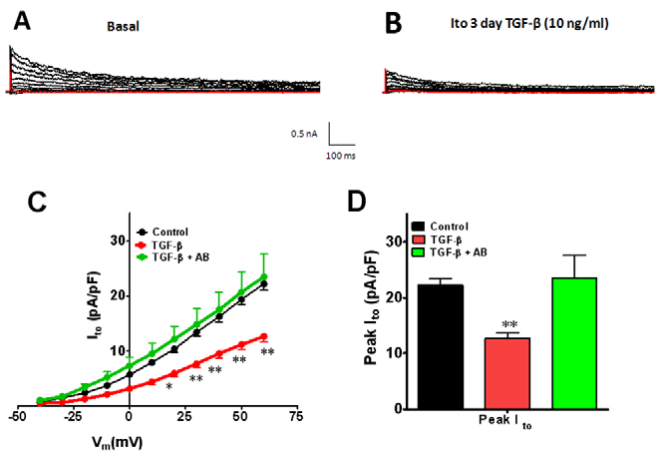
Effects of TGF-β1 on outward potassium current (I_to_) after 72 hr treatment. Representative current traces A) control, B) TGF-β1, Ito IV relation at voltages between −40 and +60 mV for control (black), TGF-β1 (red) and TGF-β1+TGF-β1 antibody (AB) is shown in panel C. D) peak I_to_ under control conditions. Values are mean ± standard error. N = 8−18 cells from 5 different isolations. *indicates p<0.05, **indicates p<0.01, ***indicates p<0.001 significant difference between control and TGF-β1 treated cells.

In our initial experiments we noted that I_to_ was somewhat less sensitive than I_Na_ to the effects of TGF-β1. Therefore, we decided to use 10 ng/ml for our experiments. Figure 5 shows that 72-hr exposure to 10 ng/ml TGF-β1 reduced the outward current to levels similar to FCM (see Fig. 2). In the presence of TGF-β1 I_to_ was significantly lower than control at voltages between +20 mV and +60 mV. At +20 mV TGF-β1 treatment reduced the current density by 42% (10.4±0.63 vs 5.9.4±0.67, p<.05); at 60 mV, current density in TGF-β1 treated cells was at 57% of control levels (22.2±1.2 vs 12.6±0.98, p<.001). The changes in I_to_ seen in TGF-β1 treated cells were completely prevented by co-treatment with the TGF-β1 neutralizing antibody (Fig. 5). A 72-hr exposure to 1 ng/ml TGF-β1 also significantly reduced the peak I_to_ but to a much modest level (data not shown).

### TGF-β1 Increases APD in Adult Cardiac Myocytes

**Figure 6:**
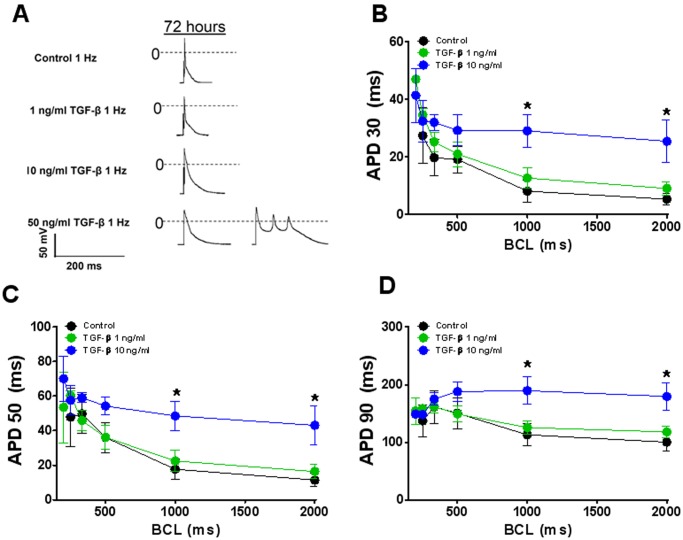
Effects of TGF-β1 on the action potential duration (APD) of ventricular myocytes at 72 hours. A) representative APs in control and TGF-β1 (1–50 ng/ml at BCL  = 1000 ms. B-D), Cycle length dependent changes in APD30, APD50, APD90 in control (black), TGF-β1 1 ng/ml (green) and TGF-β1 10 ng/ml (blue). Values are mean ± SE. N = 6−8 cells in each group. From 3 different isolations.*indicates p<0.05, significant difference between control and TGF-b1 treated cells.

From the substantial, yet contrasting effects of both FCM and TGF-β1 on the I_Na_ and I_to_ densities one would expect significant alterations in the action potential characteristics. As shown in Fig. 6, TGF-β1 (10 ng/ml) led to a basic cycle length (BCL) dependent action potential duration (APD) prolongation. For example, at a BCL of 1000 ms APD_30_ was >3.5 times larger in TGF-β1 treated cells compared to control (8.1±3.6 vs 29.1±5.6 ms; p<0.05). At 50 ng/ml TGF-β1, APD was so prolonged that some cells early afterdepolarizations (EADs).

### TGF-β1 Leads to Differential Transcriptional Regulation of Channel Protein Genes

**Figure 7:**
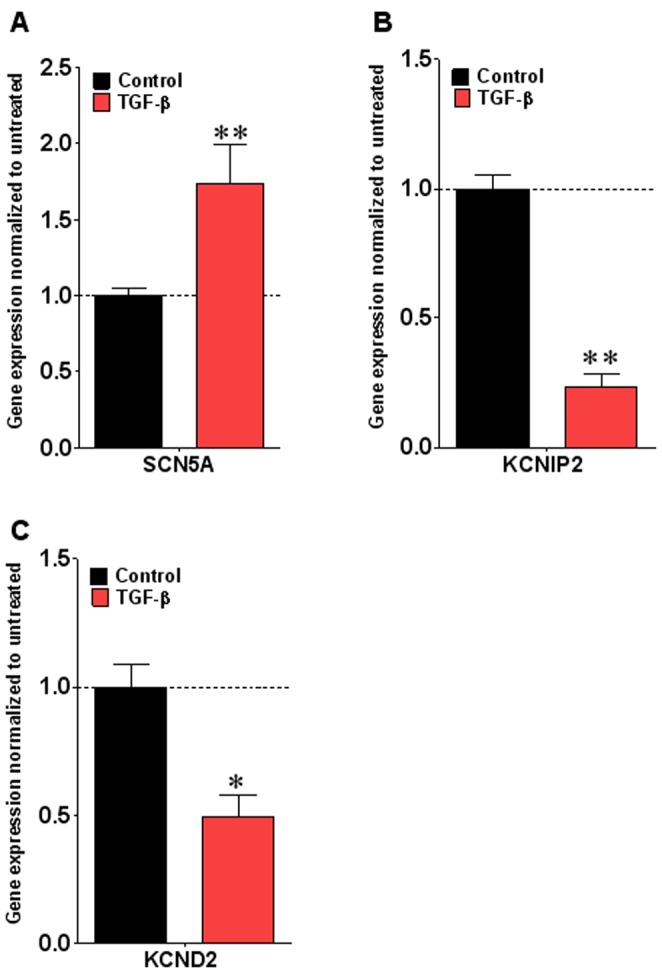
Effects of TGF-β1 on mRNA expression after 72 hr treatment with TGF-β1 (1 ng/ml). Real time PCR was performed using Taqman Primers. GAPDH was used as an internal control. A) Changes in SNC5A gene expression, B) changes in *KCNIP2* gene expression and C) changes in *KCND2* gene expression. Values are mean ± standard error of the mean, N = 6 in each group. *indicates p<0.05, **indicates p<0.01 significant difference between control and TGF-β1 treated cells by two-way ANOVA with Bonferroni post-test.

To investigate the molecular mechanism underlying the TGF-1 induced changes in I_Na_ and I_to_ densities, we used qPCR in homogenates of isolated cells after 72 hr exposure to 1 ng/ml TGF-β1. This was the concentration that achieved maximum effect on the sodium current density (see Fig. 4C). As illustrated in Fig. 7A, in accordance with the increase in I_Na_ density, *SCN5A* transcript levels were significantly increased by 1.73±0.26 fold (p<0.01). On the other hand as shown in Fig. 7B, 1 ng/ml TGF-β1 significantly reduced mRNA levels of KCNIP2 by 77% (p<0.01). Moreover, in Fig. 7C, comparison of *KCND2* expression in TGF-β1 treated cells showed a 50.6% decrease with respect to control (p<0.05).

Altogether the data presented thus far strongly suggest that the contrasting effects of TGF-β1 on I_Na_ versus I_to_ may be the result, respectively, of increased transcription and functional expression of *SCN5A* and reduced transcription of *KCNIP2* leading to reduced *KCND2* functional expression.

### Different Signaling Pathways Mediate Differential TGF-β1 Effects on ion Channel Transcription

Both NF-κB [22] and the MEK/JNK pathways [23] have been implicated in the regulation of *KCNIP2* transcription. In addition, previous work supports a link between TGF-β1 and NF-κB signaling. [24] Those data point toward NF-κB signaling as a likely regulator of the TGF-β1 induced reduction in *KCNIP2/KCND2* expression. Moreover, NF-κB has been implicated in the Ang II-induced decrease of *SCN5A* transcription and sodium current. [25] However, our data demonstrate that TGF-β1 increases Na_V_1.5 transcription and I_Na_ (Figs. 4 and 7). Thus NF-κB is unlikely to have a role in Na_V_1.5 upregulation.

### TGF-β1 Regulates SCN5A Expression via PI3K/Akt Mediated Phosphorylation of FOXO

**Figure 8:**
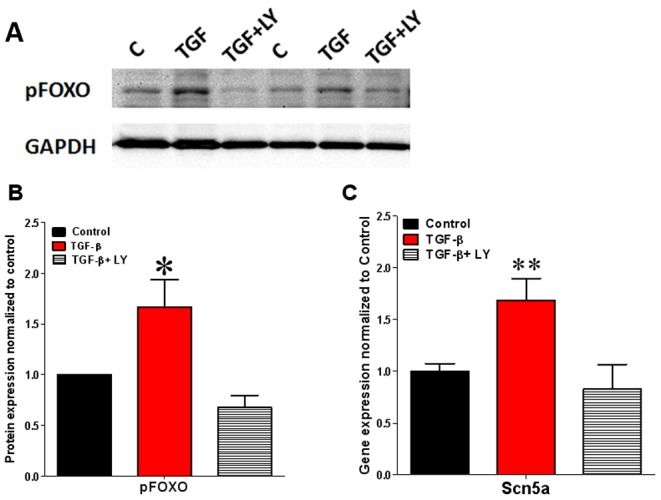
Effect of TGF-β1 treatment on FOXO1 phosphorylation in adult cardiac myocytes. A) Representative western blot image of FOXO phosphorylation by **TGF-β1** B) Quantitative data from 6 different experiments. Data are mean ± SE, N = 6. *indicates p<0.05 significant difference between control and TGF-β1 treated cells. C) Effect of LY29004 (PI3K inhibitor) treatment on SCN5A expression in TGF-β1 treated cells. Data are mean ± SE, N = 6. **indicates p<0.01 significant difference between control and TGF-β1 treated cells. LY is LY29004 (PI3K inhibitor).

We hypothesized that in the adult rat myocyte the TGF-β1 induced increase in *SCN5A* transcription occurs via a direct interaction of TGF-β1 receptors with PI3K. [26], [27] PI3K acts on membrane phosphatidylinositol (PI) to generate the second messenger lipid PI-3-4-5-triphosphate, which recruits phosphatidylinositol-dependent kinase 1 and Akt kinase to the membrane. [28] Then PI-dependent kinase-1 phosphorylates and activates Akt, which is known to phosphorylate several downstream proteins, including the Forkhead (FOXO) transcription factors, to control cell survival, cell growth and protein synthesis. [28]–[30] A recent study has indicated that FOXO1 negatively regulates *SCN5A* transcription, [31] and effect that can be inhibited by Akt induced phosphorylation and translocation of FOXO1 from the nucleus to the cytoplasm. As shown in Fig. 8A TGF-β1 (1 ng/ml) induced phosphorylation of FOXO1 in adult rat cardiomyocytes at 5 min with peak at 15 minutes exposure. In Fig. 8B, we demonstrate a 67% increase in phosphorylated FOXO1 in TGF-β1 treated cells compared to the levels in control treated cells. This increase in pFOXO1 was statistically significant (p<.05). Moreover, phosphorylation of FOXO1 in TGF-β1 treated cells was inhibited in cells pretreated with PI3K inhibitor LY 29004.

In Fig. 8C, we demonstrate that the increased activation of PI3K was responsible for the TGF-β1-induced increase in *SCN5A* transcription. On the other hand, cardiomyocytes pretreated with the PI3K inhibitor LY 29004 failed to show increased *SCN5A* transcription by TGF-β1.

**Figure 9:**
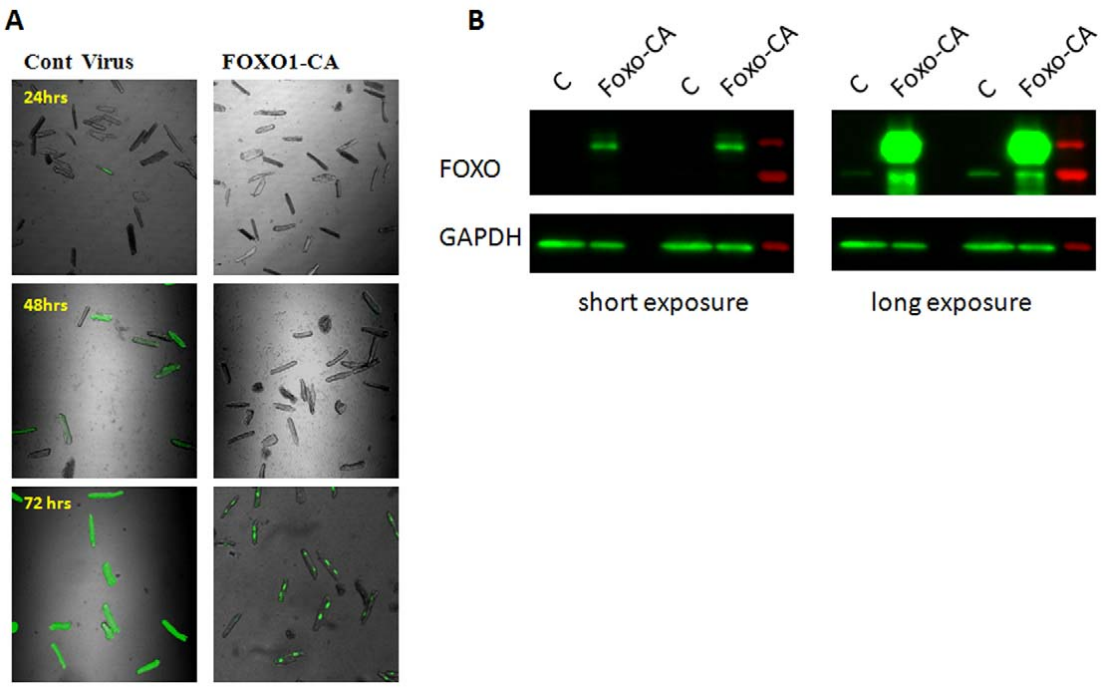
Over expression of constitutive active FOXO1 in adult rat cardiac myocytes. A) Adult rat cardiac myocytes infected with 10 MOI of virus images taken at different time after infection. B) Representative image of western blot of adult rat cardiac myocytes lysate 72 hr after infection showing increased expression of GFP tagged FOXO-CA protein.

**Figure 10:**
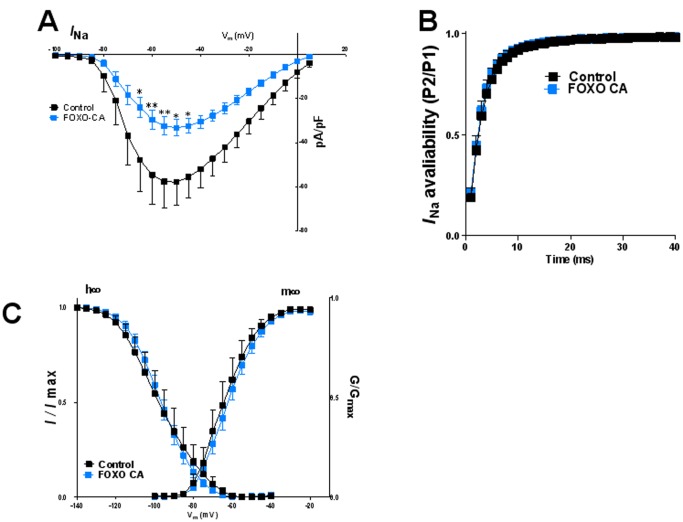
Effects of FOXO1 overexpression on peak inward sodium current (I_Na_) after 72 hr of treatment. A) Current-voltage relationships for control (black) and FOXO1-CA (blue) expressing cells. B) Time dependent recovery of the channel. Shown in panel C, Voltage dependence of activation (m∞ curve) and inactivation (h∞ curve). Values are mean ± SE. N = 9−23 cells from 4 different isolations *indicates p<0.05, **indicates p<0.01, significant difference between control and treated cells.

We further surmised that overexpression of a dephosphomimetic (Ser256>Ala), constitutively-active and nuclear-localized form of FOXO1 (FOXO1-CA) should help confirm possible repressive effects of this factor on Na_V_1.5 while over-riding its intrinsic regulation. In Fig. 9 we provide morphologic and biochemical evidence for successful virally-mediated FOXO1-CA overexpression in adult rat cardiac myocytes. In Fig. 10 we demonstrate the functional consequences of FOXO1-CA overexpression on the cardiac sodium current. As shown by the superimposed IV relation in panel A, FOXO1-CA significantly decreased the peak I_Na_ density at step voltages between −65 mV and −45 mV (p<0.05–0.01) compared with control treated cells. At −55 mV the FOXO1-CA treated cells had 43.8% less I_Na_ compared to cells treated with control treated (p<0.01). As shown in B, recovery from inactivation was not affected. Similarly, as shown in panels C, there was no change in in the voltage dependence of either activation or inactivation. We also studied the effects of virally-mediated GPF expression alone, which decreased slightly, but not significantly the peak sodium current, and shifted the m_∞_ and h_∞_ curves somewhat in the depolarizing direction (data not shown).

## Discussion

We have tested the hypothesis that TGFβ1 differentially affects cardiac ion channel function by targeting signaling pathways that modify transcription and gene expression. Our most important results may be summarized as follows: 1. Adult rat cardiomyocytes exposed for 72 hrs to FCM expressed a significantly higher peak inward I_Na_ density than control. In contrast, FCM significantly reduced peak I_to_. 2. Pre-treatment with TGF-β1 neutralizing antibody prevented the FCM-induced changes in I_Na_ and significantly reduced the I_to_ changes. 3. TGF-β1 increased I_Na_ density in a dose-dependent manner. In sharp contrast, TGF-β1 decreased I_to_ density. 4. qPCR analysis demonstrated that at 1 ng/ml TGF-β1 significantly increased *SCN5A* (Na_V_1.5), while reducing *KCNIP2* (KChip2) and *KCND2* (K_V_4.2) mRNA levels. 5. The TGF-β1-induced increase in *SCN5A* was mediated, at least in part, by activation of the PI3K-Akt pathway via phosphorylation of FOXO1. Thus, TGF-β1 released by myofibroblasts differentially regulates transcription and function of the main cardiac sodium channel and of the channel responsible for the transient outward current, with significant electrophysiological consequences.

### TGF-β1, a Major Player in Electrical Remodeling?

It is not surprising that TGF-β1 turned out to be the major cytokine responsible for the electrophysiological changes induced by long-term exposure of cardiomyocytes to FCM in our experiments. TGF-β1 is a locally generated cytokine and is the major isoform in the heart. [32] TGF-β1 contributes to myofibroblast activation and proliferation, as well as production of extracellular matrix components.[33]–[35] In addition TGF-β1 promotes fetal gene expression in cultured neonatal cardiac myocytes and is a major factor in the regulation of fibrosis and cellular hypertrophy. [11], [36], [37] TGF-β1 expression is upregulated by angiotensin II (Ang II) via activation of the angiotensin type 1 (AT_1_) receptor in cardiac myocytes and fibroblasts. [37] Induction of TGF-β1 and its mediators, including Smad proteins (Smad2, 3 and 4) and TGF-β-activated kinase-1 (TAK1), is essential for Ang II-induced cardiac hypertrophy in vivo. [37], [38] In addition, Ang II rapidly and transiently decreases K_V_4.3 mRNA expression. [39] However, to our knowledge, there are no data in the literature indicating whether TGF-β1-Smad signaling mediates the effects of Ang II on K_V_4.3 expression or whether it modifies any other channels involved in the electrical function of the adult heart. Our results demonstrate for the first time that exposing adult rat myocytes to TGF-β1 for 72 hours in culture increases I_Na_ in a dose dependent manner and reduces I_to_, and that these effects are mediated at least in part by differential transcriptional regulation of Scn5a and KChip2, respectively. These effects likely contributed to both action potential duration prolongation and development of EADs upon long term exposure to TGF-β1. However, the latter pro-arrhythmic effects need to be interpreted with caution since APD prolongation and DADs were found at BCLs longer than 500 ms, which are clearly too slow for the rat ventricular myocyte. In addition, while we only investigated the effect of FCM and TGF-β1 on Na_V_1.5, K_V_4.2 and Kchip2, it is probable that additional sarcolemmal ion channels and ion channel regulatory proteins might also be playing a role in the effects FCM/TGF-β1 treatment on APD. Also, we did not investigate effects of TGF-β1 on the late sodium current, which might have contributed to both APD prolongation and EAD formation. Nevertheless, from the foregoing we can conclude that TGF-β1 released from myofibroblasts leads to ion channel gene expression changes in the myocyte. Therefore, in addition generating an excessive ECM in the injured myocardium, myofibroblasts may modify surviving myocyte electrical activity and arrhythmogenesis and are a potential target for antiarrhythmic therapy.

### Difference in Adult vs Neonatal Cells

Long-term exposure to FCM or TGF-β1 increased I_Na_ but reduced K_V_4.2 in our experiments. These effects are almost the exact opposite of what other investigators have observed in neonatal cardiomyocytes. [14], [40], [41] For example, recently Ramos-Mondragon reported TGF-β1 reduces I_Na_ and Na_v_1.5 expression but does not affect I_to_ in neonatal atrial myocytes. Clearly these contrasting effects may be attributed, at least in part, to developmental changes in gene expression as we as in the regulation of the biophysical and electrophysiological properties of the cardiomyocytes. The action potential morphology is significantly different in neonatal compared to adult cardiomyocytes from rodents [42] suggesting major differences between the channel properties and/or associated proteins in these two cell types. For instance, in mouse cardiomyocytes the APD shortens from neonatal to adult cells and this decrease in APD is associated with increased current and expression of various potassium currents, including I_to_. In fact, K_V_4.2 protein levels are 6 times higher in adult compared with neonatal cardiomyocytes [42]. On the other hand, *I*_Na_ is smaller, activates and inactivates more slowly, and displays less negative voltage dependence of inactivation in the neonatal than adult rat cardiomyocytes. [43] These differences in phenotype have been attributed to the absence of sodium channel β1 subunit (Na_v_β1) in neonatal myocytes. Na_v_β1 is required for the development of a mature neural and cardiac sodium current phenotype. [43], [44] This raises the interesting possibility that in addition to *SCN5A*, TGF-β1 also modifies Na_v_β1 expression.

### Different Signaling Pathways Mediate TGF-β1 Effects on Ion Channel Transcription

TGF-β1 contributes to myofibroblast activation and proliferation, as well as production of ECM components. [33] In addition TGF-β1 promotes fetal gene expression in cultured neonatal cardiac myocytes and is a major factor in the regulation of fibrosis and cellular hypertrophy [11]. Most of these effects of TGF-β1 are mediated via activation of Smad proteins (Smad2, 3 and 4) and TGF-β-activated kinase-1. [37] Yet, to our knowledge, there are no previous data in in literature regarding the effect of TGF-β1 on cardiac ion channel transcription and functional expression. Our data demonstrate that TGF-β1 increases I_Na_ but reduces Ito and that these effects can be mediated at least in part by differential transcriptional regulation of *SCN5A and*, *KCND2*, respectively.

Previous work has demonstrated that both NF-κB [22] and the MEK/JNK pathways [23] regulate *KCNIP2* transcription. Furthermore, previous work supports a link between TGF-β1 and NF-κB signaling. [24]. Thus, TGF-β1 via TGF-β-activated kinase-1 may be activating JNK and/or thus translocating the NF-κB complex to the nucleus to inhibit the KChip transcription, thus reducing *KCND2* and ultimately I_to_. While we have not explore any of these two pathways in detail they offer a reasonable road map for future experiments aimed at further increasing our mechanistic understanding of the role of TGF-β1 in electrophysiological remodeling.

As discussed above, PI3K signaling leads to Akt kinase recruitment to the membrane. TGF-β1 increases Akt activation in human skin cells, an effect that is blocked by PI3K inhibition, which suggests that TGF-β1 is capable of activating the PI3K/AKT pathway. [28] Activation of Akt can phosphorylate and inactivate three of the forkhead transcription factor proteins: FoxO1, FoxO2 and FoxO4. [30] FoXO1 has a prominent role in cell proliferation and maturation. As with other cell types, myocyte-specific overexpression of FoXO1 decreased myocyte proliferation and increased maturation thus leading to embryonic lethality. [45]. It has been shown also that transcriptional activity of FoxO1 is inhibited in cardiac hypertrophy through its phosphorylation. [46] TGF-β1 increases PI3K mediated Akt activation in rat mesangial cells and induces phosphorylation and translocation of FoxO3A from the nucleus to the cytosol. [26] A promoter analysis in Cre induced downregulation of FoxO proteins showed that decrease in FoxO proteins can increase *SCN5A* transcript in a thymocyte lineage. Importantly, the promoter region of *SCN5A* encoding Na_V_1.5 has 5 FoxO1 binding sites. [47] Accordingly, FoXO1 expression in HL-1 cells decreased *SCN5A* promoter activity whereas FoxO1 siRNA increased sodium protein in these cells [31]. Here we show that TGF-β1 leads to phosphorylation of FOXO1, which likely promoted its translocation from the nucleus to the cytoplasm thus relieving its negative regulation on *SCN5A* transcription and increasing functional expression of the channel in the form of an increased I_Na_ density. We further show that by inhibiting PI3K activation by TGF-β1 the upregulation of *SCN5A* transcription is inhibited.
